# High-surface-area activated carbon from pine cones for semi-industrial spray deposition of supercapacitor electrodes†

**DOI:** 10.1039/d2na00362g

**Published:** 2022-09-21

**Authors:** Andreas Nordenström, Nicolas Boulanger, Artem Iakunkov, Gui Li, Roman Mysyk, Gaetan Bracciale, Paolo Bondavalli, Alexandr V. Talyzin

**Affiliations:** Department of Physics, Umeå University Umeå Sweden alexandr.talyzin@umu.se; Centre for Cooperative Research on Alternative Energies (CIC energiGUNE), Basque Research and Technology Alliance (BRTA) Alava Technology Park, Albert Einstein 48 01510 Vitoria-Gasteiz Spain; Thales Research & Technology 1 Avenue Augustin Fresnel 91767 Palaiseau France

## Abstract

High surface area carbons are so far the best materials for industrial manufacturing of supercapacitor electrodes. Here we demonstrate that pine cones, an abundant bio-precursor currently considered as a waste in the wood industry, can be used to prepare activated carbons with a BET surface area exceeding 3000 m^2^ g^−1^. It is found that the same KOH activation procedure applied to reduced graphene oxide (rGO) and pine cone derived biochars results in carbon materials with a similar surface area, pore size distribution and performance in supercapacitor (SC) electrodes. It can be argued that “activated graphene” and activated carbon are essentially the same kind of material with a porous 3D structure. It is demonstrated that the pine cone derived activated carbon (PC-AC) can be used as a main part of aqueous dispersions stabilized by graphene oxide for spray deposition of electrodes. The PC-AC based electrodes prepared using a semi-industrial spray gun machine and laboratory scale blade deposition of these dispersions were compared to pellet electrodes.

## Abstract 2

Activated carbon (AC) with extremely high surface area (∼3000 m^2^ g^−1^) was prepared starting from pine cones as a precursor and used as a main part of aqueous dispersions for spray and blade deposition of supercapacitor electrodes.

## Graphical abstract

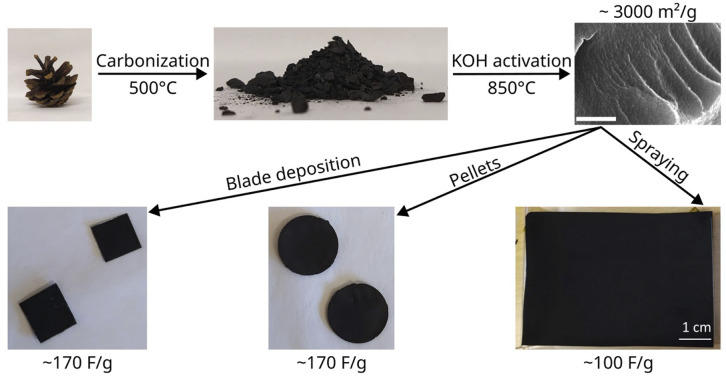

## Introduction

Electrochemical double-layer capacitors (EDLCs) are energy storage devices with high power density, long cycle life (>10 0000 charge/discharge cycles) and rapid charge/discharge. The energy density of EDLCs is lower compared to batteries.^1^ Nevertheless, EDLC-type supercapacitors (SCs) are currently used in engine starters, backup power systems, renewable energy sources and many other applications, which require high power in short intervals of time or rapid charging/discharging.

Porous and electrically conductive materials are required to prepare supercapacitor (SC) electrodes with high energy storage parameters. High surface area carbon materials (2000 m^2^ g^−1^) are the key elements of modern commercial SCs. A high specific surface area (SSA) translates, up to a certain extent, to more electrostatic charge storage. The most common high surface area carbon material is microporous activated carbon (AC). There have been many reports focusing on the preparation of low-cost AC from various bio-materials including various kinds of waste (*e.g.* old tires,^2^ food waste,^3^ and spent tea leaves^4^) and agricultural wastes (*e.g.* corn cobs,^5^ coconut shells,^6,7^ and sawdust^8^). Converting waste into useful materials contributes also to resolving environmental issues related to waste recycling. A variety of activation methods were proposed to convert biochars into AC, each with own advantages and drawbacks. Possibly the most common in industrial applications is steam activation typically providing AC with a surface area of about 2000 m^2^ g^−1^.^7,9,10^ However, a much higher surface area (>3000 m^2^ g^−1^) was reported for AC carbons produced by KOH activation.^11^

Many kinds of nanostructured carbon materials and composites have been tested as electrode materials on the laboratory scale in recent years.^12–15^ Activated reduced graphene oxide (a-rGO) or “activated graphene” is a promising material for the preparation of SC electrodes, due to a combination of a high SSA (∼3000 m^2^ g^−1^), high conductivity and mechanical stability.^16,17^ Gravimetric capacitance values in the range of 90–300 F g^−1^ have been reported for supercapacitors prepared using a-rGO^16–19^ with some variation of precursors, modification of the structure and pore size distribution of these materials.^20–22^

It should be noted that the performance of SCs depends not only on the properties of porous materials but also on the methods of electrode preparation, *e.g.* due to influence of added components and specifics of deposition. Spray-gun deposition of electrodes was demonstrated in recent years as a feasible method to prepare large area electrodes for SCs on the semi-industrial scale.^23–26^ Recently, we have demonstrated that stable aqueous dispersions can be prepared using micrometer sized a-rGO grains and graphene oxide (GO) as a stabilizing agent.^27^ The dispersion also included fumed silica as a rheology modifying component and carbon nanotubes (CNTs) in order to improve electrical contact between a-rGO grains in electrodes. Thermal annealing was demonstrated to further improve performance of SC devices due to conversion of electrically insulating GO into conductive rGO. The dispersions have been prepared in liter amounts demonstrated to be sufficiently stable for deposition using industrial spray deposition.^28^

Activated graphene is a promising material for SCs, but it is relatively expensive due to the high cost of the precursor rGO. It should be noted that a-rGO is prepared by the KOH activation method, which is also commonly used to prepare AC. Both a-rGO and AC are carbon materials with a 3D porous structure. The main difference between “activated graphene” and AC is the type of precursor used for the activation. Reduced GO is used to produce “activated graphene”, and most commonly, various biochars are used to prepare AC. It is not yet clear if using rGO as a precursor provides significant advantages in terms of supercapacitor performance since the 2D sheets are anyway connected into a rigid 3D structure in the process of activation. Preparation of rGO is also a relatively complex process. It starts with oxidation of graphite into graphite oxide, which involves using strong acids, slow adding of an oxidant and thermally controlled conditions. The graphite oxide is then subjected to explosive thermal exfoliation to produce rGO. Biochars are a lot easier to prepare starting from variety of organic precursors using simple thermal annealing procedures. Therefore, we decided to prepare AC by exactly the same KOH activation procedure as the one previously used in our earlier studies^27,28^ to prepare “activated graphene” but using natural biochar instead of rGO as a precursor. We also evaluated the performance of these ACs in SC devices.

Pine cones were selected as a starting material for preparation of AC due to its high abundance in Scandinavia (also in whole Europe and North America) and reports of extremely high surface area materials (up to ∼3900 m^2^ g^−1^) obtained using KOH activation of pine-cone based biochars.^29–33^ Activated carbon from pine cones has been studied previously as a bio-sorbent for metals, CO_2_, dye waste, nitrate, and removal of lead(ii) ions from aqueous solutions by adsorption.^8,29,31,33–36^ There are relatively few reports on the use of pine cone derived carbons for the preparation of SC electrodes.^32,37–41^ However, most of these studies were performed using AC with a SSA (1000–2000 m^2^ g^−1^) significantly lower than that of a-rGO and prepared by somewhat different procedures^32,38,41^ with a single report on the material with ∼2500 m^2^ g^−1^ which is still below the surface area of a-rGO (up to ∼3000 m^2^ g^−1^).^37^

Moreover, an extremely high BET SSA (*e.g.* 3900 m^2^ g^−1^ in ref al^29^) of pine cone derived AC (PCAC) was not always evaluated using verified procedures specific for microporous materials designed by J. Rouquerol *et al.*^42^ and requires independent verification. The value of the BET SSA of microporous materials is particularly dependent on the selection of the relative pressure (*P*/*P*_o_) range for the BET plot and can easily be extracted erroneously with rather different values from the same N_2_ isotherm. The correct procedure, which allows reading reproducible values of the SSA in microporous materials, includes a special method to select the right interval of relative pressures for a BET plot.^42^ The SSA value is also more reliably determined using suitable DFT models (providing typically smaller numbers). Moreover, evaluation of pore size distribution in microporous materials requires rather detailed isotherms with many points at a low relative pressure interval which was not always the case in published studies.

In this study we produced AC with an exceptionally high BET surface area of ∼3000 m^2^ g^−1^ using pine cones as a “green” precursor typically considered as a waste in the wood industry. The performance of AC was verified in SC devices prepared using three different electrode preparation methods, such as using compressed pellet electrodes, blade deposition and semi-industrial spray-gun deposition using stable aqueous dispersions with graphene oxide as a stabilizing agent. Our results demonstrate that using the same KOH activation procedure with rGO or pine cone derived biochars provides porous carbon materials with similar SSA values, pore size distribution and electrode performance in EDLCs. Therefore, pine cone-derived AC can be considered as an inexpensive and “green” alternative to “activated graphene” (a-rGO) in dispersions used for spray deposition of SC electrodes.

## Experimental

**Fig. 1:**
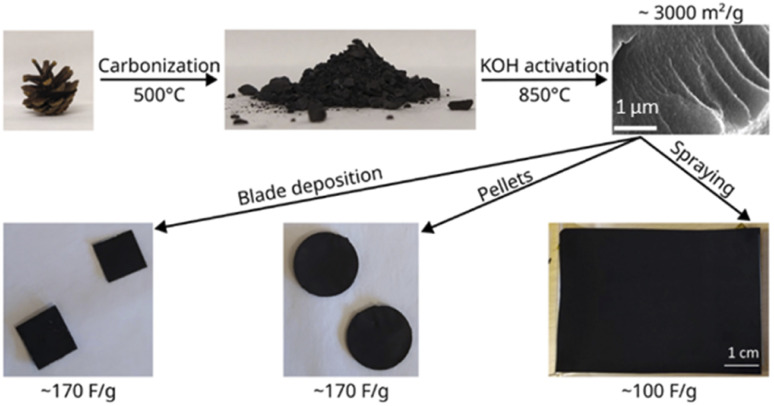
The scheme of the study illustrating synthesis of activated carbon starting from pine cones, preparation of electrodes and gravimetric capacitance for electrodes prepared using three different methods.

The scheme of this study is presented in Fig. 1 which illustrates the synthesis of the material and characterization in supercapacitor devices. The experimental details for each step are provided below.

### Carbonization of pine cones

The pine cones were collected on a sunny day in a forest outside of Umeå, Sweden. The site was far from the city and industry to minimize the risk of inorganic contaminants in the pine cones. Pine cones were washed several times in DI water and dried at 70° for a few days. The dry pine cones were carbonized in a tube furnace by heating from room temperature to 500 °C with a rate of 5 K min^−1^ and keeping at 500 °C for 2 h under the flow of argon. This carbonization temperature program was chosen based on a previously reported procedure.^29^ The carbonized pine cones (biochar) were crushed into fine powder using an agate mortar before the activation procedure.

### Activation procedures

The biochar produced from pine cones was activated using the KOH procedure optimized in our earlier publications for the rGO precursor to produce the highest BET surface area.^17,43^ The biochar powder was mixed with KOH in a 1 : 8 proportion by weight and placed in a water/ethanol mixture (3 : 7 by volume). Typically, we used 50 ml of the water/ethanol solution for every 1 g of biochar. The mixture was magnetically stirred overnight and dried in a vacuum oven. The dry powder was then annealed under argon flow using the following temperature profile:

- Heating from 25 °C to 200 °C in 20 min (8.75 °C min^−1^).

- Annealing at 200 °C for 30 min.

- Heating from 200 °C to 850 °C in 2 h 20 min (4.6 °C min^−1^).

- Annealing at 850 °C for 3 h.

- Cooling back to ambient temperature (5–6 hours with the furnace switched off).

The reaction product was washed with 10% aqueous solution of acetic acid, filtered using a PTFE filter, then washed with water (until the pH was in the range 4–6) and dried.

The carbonization step from dry pine cones to biochar has a yield of 32.2 wt%, and the activation step has a yield of 32.5 wt%. The amount of dry pine cones used was 16.2 g, which yielded 1.3 g of activated material, which corresponds to 10.5 wt%.

A separate batch of activated pine cones was prepared according to the procedure described by K. Li *et al.*^29^ which was reported to result in AC with the highest SSA (3931 m^2^ g^−1^). KOH was grinded in a mortar and mixed with dry biochar (2 : 1 by weight). The mixture was then placed in a tube furnace and heated from room temperature to 800 °C with a heating rate of 3 K min^−1^, followed by 1 h annealing at 800 °C before cooling down to room temperature. The reaction product was washed with 10% HCl solution with stirring for 5 min and then rinsed with water before drying at 105 °C for 3 h. However, analysis of AC produced using this procedure showed a BET SSA value of ∼1850 m^2^ g^−1^, much smaller than expected from the report and our own procedure described above. For this reason, the procedure described by K. Li *et al.*^29^ was not used in our experiments with electrode preparation.

### Characterization of materials

The as-synthesized AC was characterized using X-ray photoelectron spectroscopy (XPS), X-ray diffraction (XRD), Raman spectroscopy, thermogravimetric analysis (TGA), SEM imaging, BET analysis and Fourier-transform infrared (FTIR) spectroscopy. XPS spectra were recorded with a Kratos Axis Ultra electron spectrometer equipped with a delay line detector. A monochromatic Al Kα source operated at 150 W, a hybrid lens system with a magnetic lens, providing an analysis area of 0.3 × 0.7 mm, and a charge neutralizer were used for the measurements. The binding energy scale was adjusted with respect to the C 1s line of aliphatic carbon, set at 285.0 eV. All spectra were processed with the Kratos software. TGA was performed by using a Mettler Toledo TGA/DSC1 STARe System. Experiments were performed from room temperature up to 700 °C at a heating rate of 3 K min^−1^ under nitrogen or air flow (40 mL min^−1^). SEM images were collected using a Zeiss Merlin FEG-SEM microscope. A Panalytical X'pert diffractometer was used to record XRD patterns in the reflection mode with CuKα radiation. Raman spectra were recorded using a Renishaw Invia Raman spectrometer equipped with a 514 nm laser. Fourier transform infrared (FTIR) spectra were recorded using powder samples using a Bruker IFS 66v spectrometer.

The nitrogen sorption isotherms were measured using an Autosorb iQ XR surface area & pore size analyzer (Quantachrome) equipped with a turbo pump at liquid nitrogen temperature. The relative pressure interval *P*/*P*_0_ for the BET plot was selected using a procedure optimized for microporous materials. The procedure takes into account Rouquerol parameters and is provided as a part of standard Quantachrome ASiQWin software package.^42^ The value of the BET SSA was also verified using the BETSI software^44^ which provided nearly identical values (see Table 1 in the ESI† file). The slit-pore QSDFT equilibrium model was applied to evaluate the cumulative surface area, pore volume and pore size distribution.

## Electrode preparation

### Preparation of electrodes using aqueous dispersions

Pine cone activated carbon (PC-AC) powder was dispersed in water with additives (graphite oxide, fumed silica, and carbon nano-tubes (CNTs)) according to the optimized component ratio 10 : 1 : 1 : 1 (activated pine cone : GO : SiO_2_ : CNT).^27^ In some samples (for blade deposition), an increased amount of GO was also tested in the proportion (10 : 2 : 1 : 1). Electrodes were obtained by simply drying the dispersion on suitable current collectors (steel substrate or aluminium). As was shown in our previous work, the electrodes prepared from this dispersion maintain a high SSA of the precursor material, which is useful for SC applications.^27^ To further improve the mechanical stability and conductivity of the electrodes, we subjected them to annealing at 200 °C for 1 h. Typically, the concentration of the dispersion used in spray deposition was 2.5 mg ml^−1^.

For blade deposition, we prepared a dispersion with a higher concentration of PC-AC (20 mg ml^−1^).

The dispersions were gun-sprayed on carbon coated aluminium foil (En’Safe Primed by ARMOR) collectors with a thickness of 20 μm. The spray gun machine has a moving spray-nozzle and maintains a constant distance of ∼15 cm from the surface. The collectors are placed on a hotplate heated up to a temperature above the boiling point of water (>100 °C) to ensure the instantaneous evaporation of the solvent droplets.^45,46^ The spray-gun is moved in the *X*–*Y* plane, and the nozzle height over the substrate can be finely adjusted. The deposition is taking place in lines with a length of 30 cm and with a pitch of around 1 cm to allow the best recovery of the sprayed cones^47^ on a total width of 10 cm. The precision for all the movements is around 100 μm. A nozzle speed of 2 cm s^−1^ and a nozzle overture of 1.3 mm were used. The carrier gas for nebulization of the suspension is nitrogen. The process allows to fabricate several electrodes using the same deposition parameters and nearly the same mass. The mass of electrodes can change as a function of the sprayed dispersion composition. In order to verify the exact amount of material deposited, current collectors are carefully weighted (PerkinElmer AD-4 autobalance with an accuracy of ∼0.02 mg) before and after spraying the graphene based dispersion.^47^ The dispersions stored for several days before deposition were mildly sonicated to improve stability and uniformity of the concentration between the top and the bottom of the bottle. We also used magnetic stirring to keep the suspension more stable during the spray deposition process.

### Preparation of pellet electrodes

A separate set of electrodes were prepared as pellets, according to the procedure described in our previous work.^48^ AC-PC powder was mixed with poly(tetrafluoroethylene) in a weight ratio of 8 : 1, and some ethanol was added. The ethanol-wetted paste was compressed in a piston cylinder cell at 50 MPa to make 12 mm diameter pellets with a typical thickness of 130–200 μm. To ensure a homogeneous thickness of the pellets, the pressure loading–release cycle was applied nine times. Then the electrodes were dried in a vacuum oven for 1 h at 200–250 °C. The annealing results in conversion of GO into rGO which improves the electrical conductivity of electrodes.

### Electrochemical characterization of supercapacitors

Electrochemical characterization of electrodes was performed using a potentiostat (Autolab PGSTAT204 or Iviumstat XR) and a standard two-electrode cell. For each measurement, two square electrodes were cut from a larger sheet (except for pellet electrodes) and weighed. Mass of the coating per unit mass of the current collector was measured by scraping off and weighing a reference sample with and without the coating (not necessary for pellet electrodes). Typical electrode sizes were 1.3–1.5 cm^2^ for electrodes measured in aqueous electrolyte and 0.5–1 cm^2^ for electrodes measured in organic electrolyte. The typical electrode mass loading was 2–3 mg cm^−2^ for the electrodes made from drying dispersion and 1.4–1.8 mg cm^−2^ for spray deposition. The typical weight of pellet electrodes was 5–7 mg. Glass fiber membranes (Whatman GF/A) were used as separators for all measurements. CV curves were measured in the voltage range 0–1 V for aqueous electrolyte and 0–2.7 V for organic electrolyte using scan rates of 50, 100, 200 and 500 mV s^−1^. Charge–discharge curves were measured in the voltage range 0–1 V for aqueous electrolyte and 0–2.7 V for organic electrolyte at specific current values ranging from 0.5 A g^−1^ up to 100 A g^−1^.

## Results and discussion

### Characterization of pine cone derived activated carbon

Activation of biochar produced from pine cones resulted in the formation of a microporous carbon material. An extremely high BET SSA of pine cone activated carbon (PC-AC) was reproduced well in three synthesis batches (2850 m^2^ g^−1^, 3047 m^2^ g^−1^ and 3048 m^2^ g^−1^). An example of the BET plot composed of 7 points in the range of *p*/*p*_o_ of 0.124–0.274 selected using the Rouquerol procedure is shown in Fig. 1S (see also Table 1 in the ESI† file).^42^ The surface area was also estimated using the QSDFT slit pore model which is a more modern method arguably providing more realistic values and not affected by somewhat subjective procedures typical for the BET method. The cumulative SSA of PC-AC for was found to be 2116 m^2^ g^−1^, 2249 m^2^ g^−1^ and 2280 m^2^ g^−1^ for batches 1,2 and 3 respectively.

**Fig. 2:**
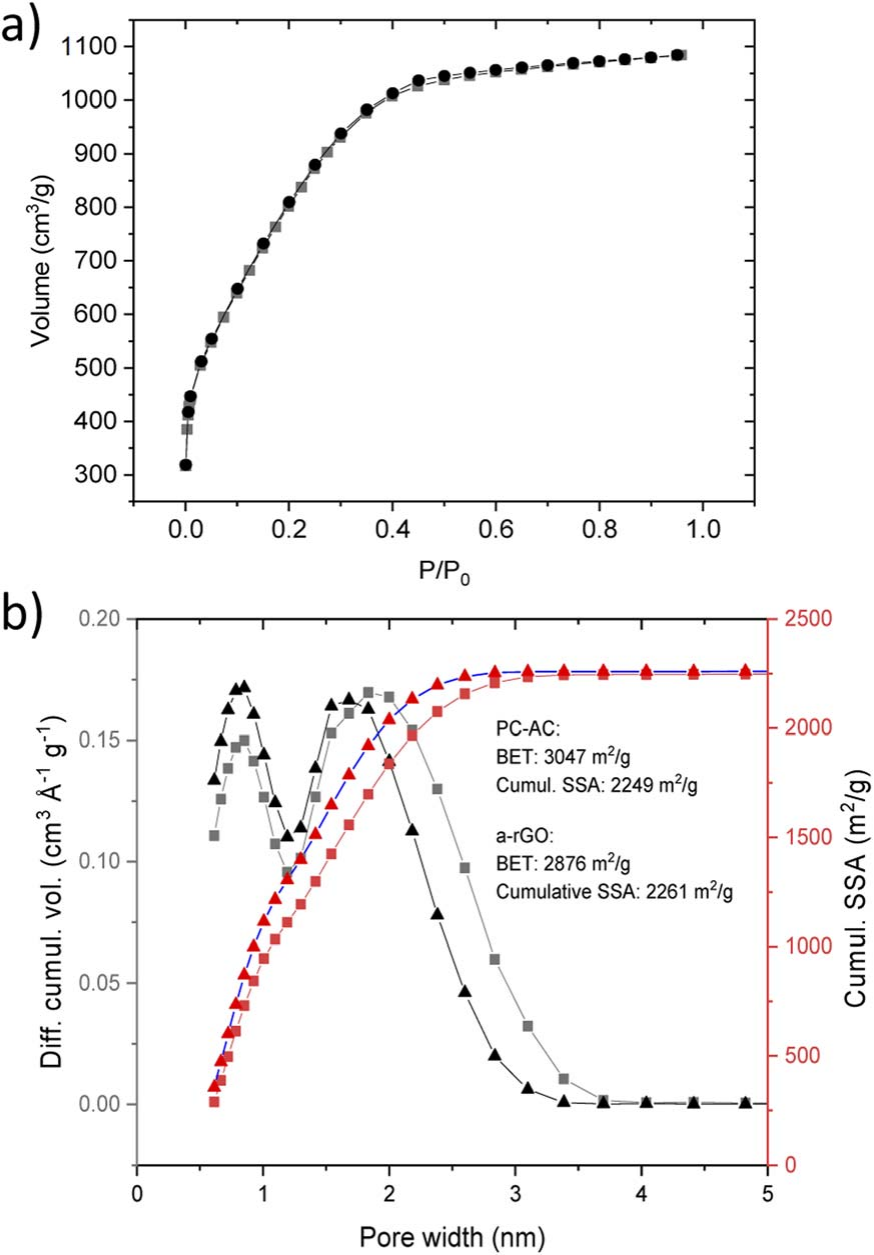
(a) Nitrogen sorption isotherm of the PC-AC sample. (b) Pore size distribution and the cumulative SSA obtained using analysis using the QSDFT mode for the PC-AC sample and sample of a-rGO (ref. 43). The PC-AC and a-rGO materials were prepared using a similar KOH activation procedure.

A somewhat smaller SSA in the first batch was most likely due to contamination with non-carbon impurities. Analysis of the N_2_ sorption isotherm of batch 3 (Fig. 2a) using the QSDFT method showed a pore size distribution (Fig. 2b) with two major peaks corresponding to diameters of ∼0.8 nm and ∼1.8 nm and all the pore volume in pores with a diameter below 4 nm.

The SSA values and pore size distribution of PC-AC are very similar to the data reported earlier for some samples of a-rGO (Fig. 2b).^43,48^ It should be noted that pore size distribution in a-rGO can be somewhat different depending on KOH loading and temperature of activation. These conditions were not completely identical in the two samples shown in Fig. 2b but rather similar. Nevertheless, the similarity of pore size distribution shows that the KOH activation procedure is the main factor which defines the resulting porosity of the materials, while the influence of the precursor (rGO or biochar) is smaller.

**Fig. 3:**
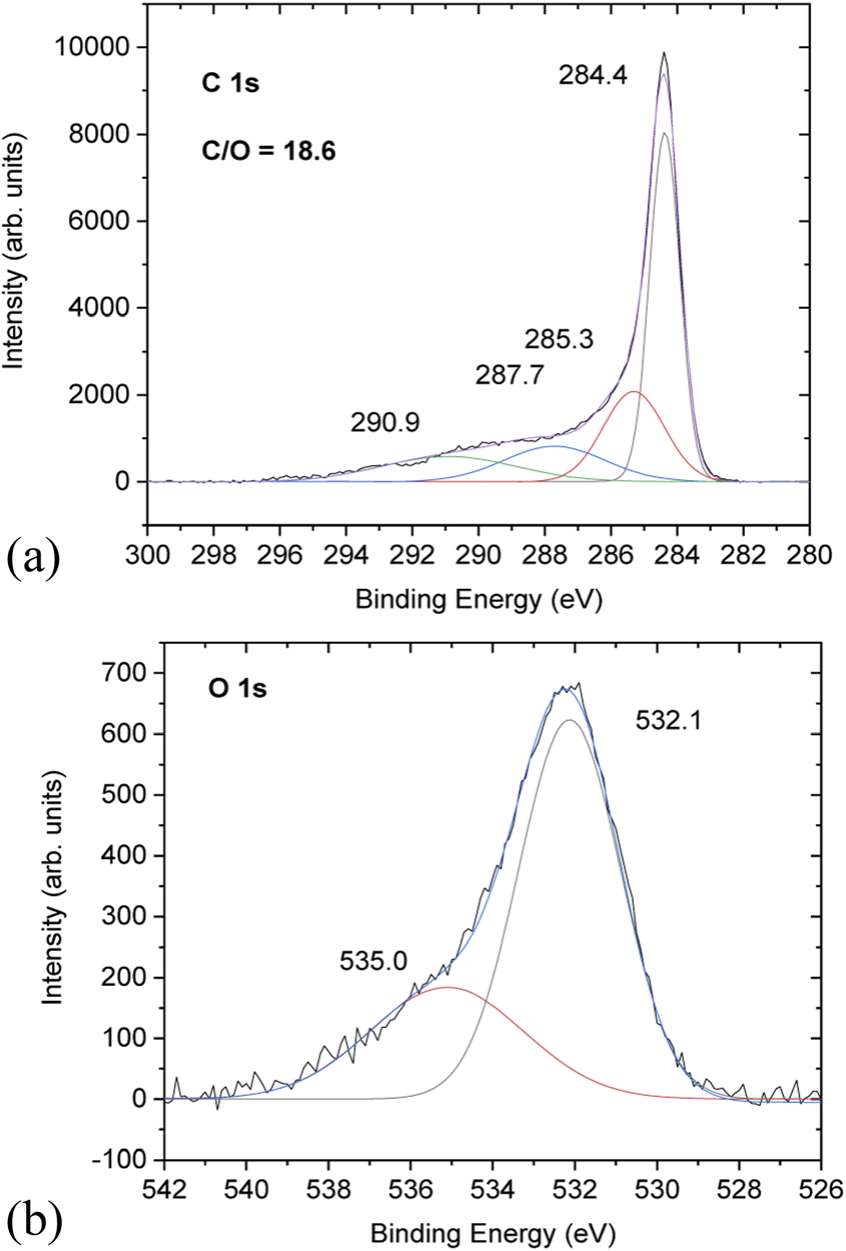
XPS spectra of PC-AC: the (a) C 1s region and (b) O 1s region.

Chemical analysis of PC-AC was performed using XPS (Fig. 3) which showed trace amounts of potassium and aluminium (from Al_2_O_3_ of the furnace tube or sand particles) impurities. Considering that a natural source was used for the preparation of PC-AC, the samples showed a rather small amount of impurities as confirmed by XPS, which revealed C/O ratios of 10.1 (batch 1) and 18.6 (batch 3).

Higher oxygen content (C/O = 10.1) correlates with the increased amount of Al_2_O_3_ in this sample. Excluding oxygen from the Al_2_O_3_ impurity, the C/O ratio increases up to a value of 67.

The C 1s part of the XPS spectra shows the standard for AC components at 284.4 eV (not oxidized carbons), 285.3 eV and 287.7 eV from carbons connected to oxygen functional groups (single-bonded and double bonded, respectively) and the satellite 290.9 eV component.

**Fig. 4:**
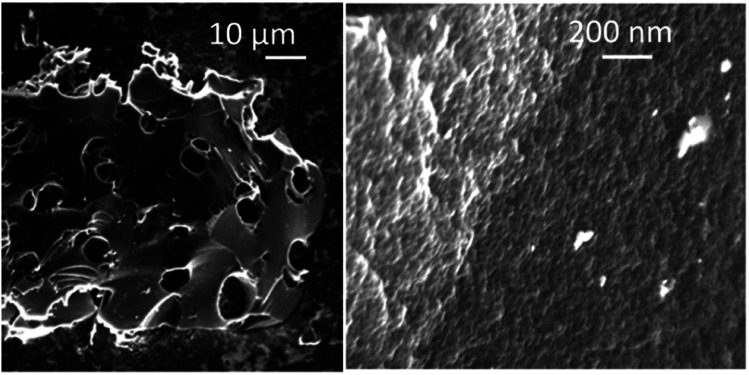
SEM images recorded from the PC-AC sample.

The PC-AC samples consist of grains with size in the range ∼1–100 μm, which are visibly porous only in the high resolution images (Fig. 4), as expected from the pore size distribution determined using the analysis of the N_2_ isotherms. Once again, the PC-AC material shows a strong similarity with earlier reported a-rGO in microscopic texture revealed by SEM. The only difference which was observed in the SEM images is the absence of clear layered textures typically found in the a-rGO grains.

As expected, the PC-AC samples are nearly amorphous according to XRD (Fig. S2 in the ESI† file) exhibiting no trace of a graphitic structure. The highly disordered structure of PC-AC is evident also from the Raman spectra which exhibit the standard for activated carbons D-and G-modes and an intensity ratio *I*_D_/*I*_G_ of about 5.64 (Fig. S3 in the ESI† file). The amorphous nature of PC-AC is expected for materials with a very high surface area. Graphitic carbon has a too small distance between graphene layers (∼3.4 Å) and not accessible for penetration of nitrogen. Therefore, graphitic regions do not contribute to sorption of nitrogen and surface area values determined using sorption. As expected, only rather weak peaks in the FTIR spectra are found due to low oxygen content (see Fig. S4 in the ESI† file). The samples were also analyzed using TGA/DSC scans performed in air and under the flow of nitrogen. These methods confirm that PC-AC has low impurity levels with only about 5% ash content not removed by heating in air (Fig. S5 in the ESI† file).

Summarizing the material characterization part, PC-AC shows a BET and DFT surface area, pore size distribution and chemical composition nearly identical to “activated graphene” prepared earlier using the identical KOH activation procedure. However, using pine cones as a precursor is clearly a much less expensive alternative compared to rGO required for the synthesis of “activated graphene”. Below, we also demonstrate that PC-AC can be used for preparation of electrodes providing the performance of supercapacitors with energy storage parameters on the same level as “activated graphene” (a-rGO).

### Testing PC-AC in supercapacitor electrodes

Electrodes for testing in supercapacitors were prepared using three methods. The first two methods involved preparation of aqueous dispersions with PC-AC as a main component. The dispersions included graphene oxide as the surfactant, fumed silica as a rheology modified agent, CNTs for better mechanical stability and improved electrical contact between with PC-AC as a main component which contributes to a high surface area of the electrode. The SSA of electrodes prepared from AC is about 20% smaller compared to the SSA of precursor material due to added components.^27^

The dispersions were spray-coated on carbon plated aluminum foil and evaluated as electrodes in SC devices using TE-BF_4_/acetonitrile electrolyte. These results were compared to tests with electrodes deposited by blade deposition and tests with pressurized pellet electrodes.

**Fig. 5:**
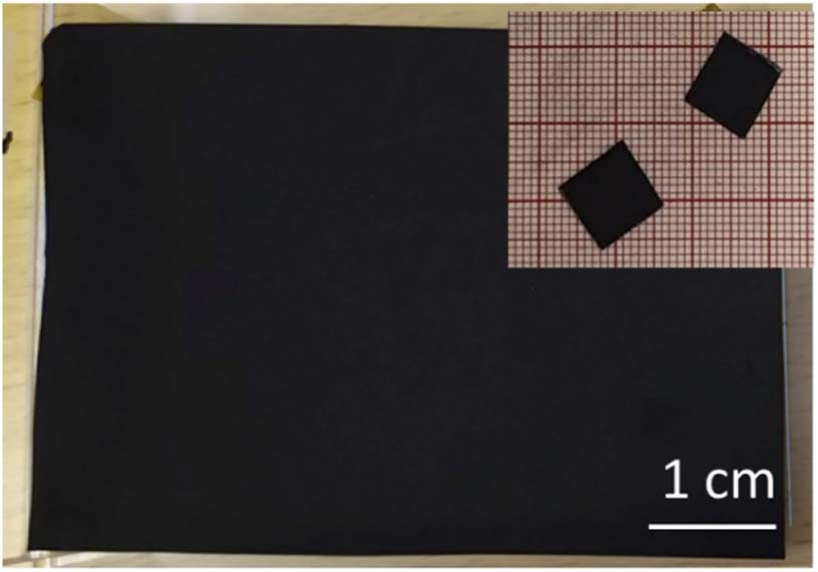
Optical image showing a piece of the spray deposited electrode and smaller pieces cut for the preparation of the SC.

The PC-AC dispersions were prepared in 1 L amount to enable several spray deposition tests using the same batch. The dispersion was black and not transparent. Visual observations demonstrated that the dispersion was stable with little precipitation on the bottom of bottles even after 2–3 days of shelf storage. The spray tests with 250 ml volume were performed using parameters optimized in our previous study for a-rGO and porous carbon dispersions.^28^ The parameters which had to be optimized include the nozzle speed, dispersion concentration, flow speed and temperature of the substrate. Visibly uniform deposition of electrodes with a mass loading of 3.22 mg cm^−2^ was achieved (Fig. 5). The electrodes were sufficiently well adhered to the current collector.

The conductivity of spray deposited electrodes was verified using small pieces separated from the current collector providing values on the level of ∼50 S m^−1^. These values were compared with the samples of AC mixed with the binder in a similar proportion providing a much smaller value of ∼0.13 S m^−1^. The increase in conductivity of spray coated electrodes is likely connected to the added components rGO and CNTs which provide electrical contacts between the grains of PC-AC (Table 2 in the ESI†).

**Fig. 6:**
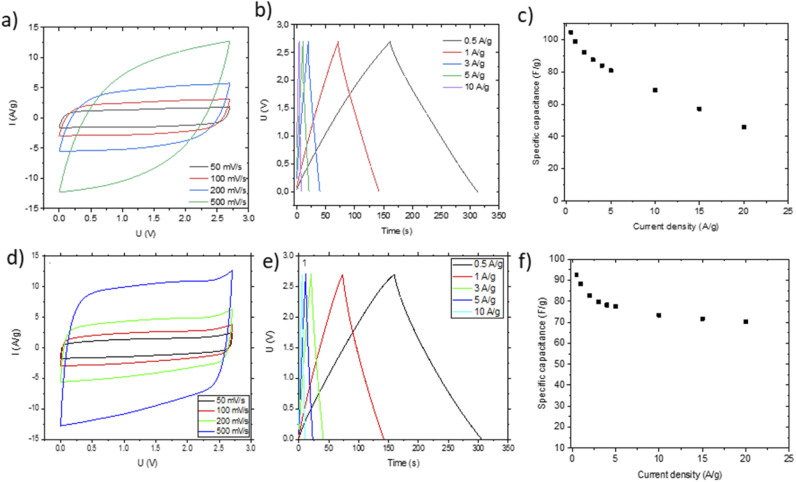
Electrochemical characterization of SC devices in TEA-BF_4_/Ac electrolyte prepared using AC:GO:CNT:SiO_2_ dispersions with spray deposited electrodes (a–c) and blade deposited electrodes (d–f). Cyclic voltammetry (a and e), charge–discharge (b and f) and specific capacitance *vs.* current density plots are shown.

Small pieces were cut from larger area foil, assembled into a SC and tested in TEA-BF_4_/acetonitrile electrolyte (Fig. 6a–c). The data obtained using SCs with electrodes prepared by blade deposition are summarized in Fig. 6d–f.

The data show a similar performance for electrodes deposited by spray and blade methods. The shape of the CV curves is close to rectangular as expected for EDLCs. However, at high scan rates, the deviation from the rectangular shape is somewhat stronger for spray deposited electrodes, and the charge–discharge curves show a shape closer to linear. Analysis of the charge–discharge plots was used to calculate gravimetric capacitance. The maximum capacitance of 105–100 F g^−1^ was found for spray-deposited electrodes and a somewhat smaller value of 93 F g^−1^ for the cell with blade-deposited electrodes at 0.5 A g^−1^ (Fig. 6). In both cases, gravimetric capacitance drops significantly at a higher current density.

The maximal capacitance of PC-AC is clearly improved compared to standard commercial Kuraray AC, which was used in our previous studies (84 F g^−1^ (ref. 28)). The improvement is likely connected to a higher SSA value of PC-AC (3050 m^2^ g^−1^) compared to 2580 m^2^ g^−1^ for Kuraray AC. However, it is still somewhat lower compared to a-rGO which showed 130 F g^−1^ in 1 M TEA-BF_4_/Ac electrolyte.

The advantage of spray deposition is that the thickness of electrodes can be increased using multiple runs over the surface of a current collector. Adding an additional layer by blade deposition typically destroys the sample. It should be emphasized that spray deposition was performed using the semi-industrial scale over a relatively large area. This method can easily be scaled up for industrial production as compared to laboratory scale handmade blade deposition.

The performance of PC-AC was also verified using pellet electrodes prepared by high pressure compaction. In this case, the electrodes do not include graphene oxide and fumed silica as a part of formulation but require a binder (PTFE in this case). As a result, the weight fraction of PC-AC is almost the same as in dispersions, but the thickness of electrodes can be made significantly larger.

The gravimetric capacitance of pellet electrodes was found to be in good agreement (considering the significantly larger thickness of pellets) with values obtained using electrodes prepared from sprayed and bladed dispersions providing the highest value of 90 F g^−1^ at 0.5 A g^−1^.

**Fig. 7:**
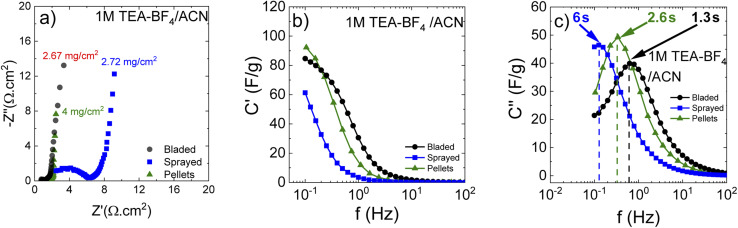
Impedance response of materials in organic electrolyte 1 M TEA-BF_4_/ACN depending on the electrode preparation method. (a) Nyquist plots also indicating mass loading; (b) real and (c) imaginary capacitance components according to the complex capacitance model showing the characteristic response time corresponding to the knee frequency.

The Nyquist plots (Fig. 7a) show that the cells prepared using sprayed electrodes display a high-amplitude semicircle related to interface resistance. This interface resistance is most likely due to the higher level of surface oxygen in the samples. The cells based on the bladed and pellet electrodes show a much lower contact resistance and behave similarly in the whole measured frequency range.

Impedance data can also be treated in terms of the frequency-dependent complex capacitance that can be used to know the efficiency of a capacitive rate response.^49^Fig. 7b shows that the sprayed electrodes are the least efficient since the real capacitance component is lower than for the bladed and sprayed electrodes at the same frequency. The bladed electrodes show the most efficient capacitive response according to their highest capacitance values among the three samples. The impedance data can also be represented as an imaginary capacitance plot, which peaks at a knee frequency (or its reciprocal characteristic capacitor response time *τ*) separating the domination of capacitance and resistance. In agreement with Fig. 7b, the bladed sample shifts to the prevailing capacitive response at the lowest characteristic time of 1.28 s whereas the pelletized electrodes show a marginally slower response.

The sprayed sample is efficiently capacitive only in the lower frequency domain (the higher characteristic response time).

As can be seen, annealing and preparation conditions together with the mass loading are all determining factors for the efficiency of a capacitive rate response. The bladed and pelletized electrodes provide a similar cell response in our samples. The increase in the overall resistance for the sprayed electrodes (Fig. 7a) slows down the capacitive response of the corresponding cells, probably due to less good electrical contact between individual grains originating from each droplet.

**Fig. 8:**
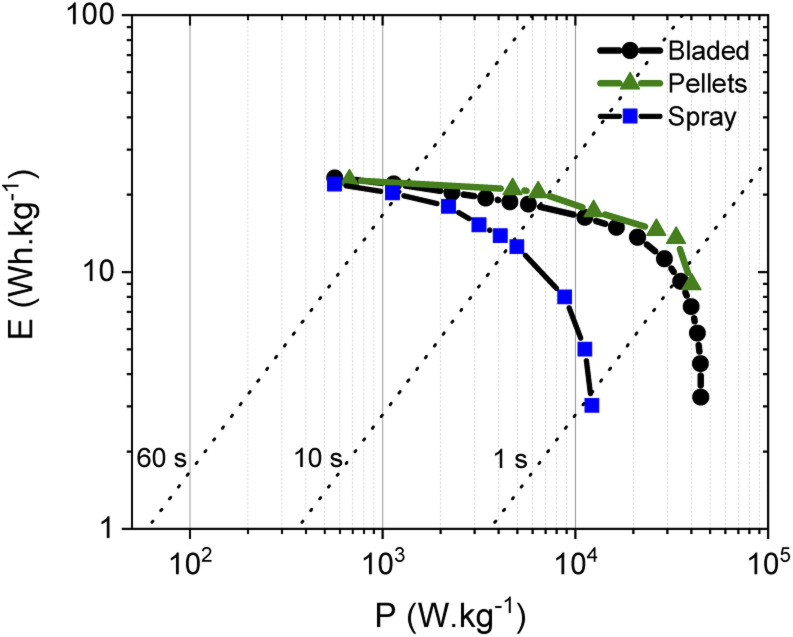
Ragone plots for SC with PC-AC electrodes prepared using: spray deposition (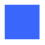), blade deposition (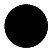) and pellet electrodes (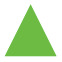) and tested in TEA-BF_4_/Ac electrolyte.

The energy storage parameters of the SC with spray deposited electrodes are similar to those made with pellets as confirmed by the corresponding Ragone plots (Fig. 8). As expected, tests performed in organic electrolyte show higher energy density compared to the measurements conducted in aqueous electrolytes (see below). This is due to the use of a wider potential window for the charge–discharge of the electrodes, as measurements in aqueous electrolyte were performed in a 0–1 V interval, while a 0–2.7 V interval was used for tests in organic electrolyte. As expected from the data shown above, the energy and power density of the SC with PC-AC electrodes is somewhat lower compared to those of a-rGO electrodes studied in our earlier work but on a comparable level.^28^

It is an interesting observation considering that PC-AC and a-rGO materials showed a very similar surface area and essentially a microporous nature. This means that some other parameters are important in the overall performance of electrodes such as the nature of the remaining oxygen groups and differences in defects derived from different precursors after essentially similar activation procedures.

**Fig. 9:**
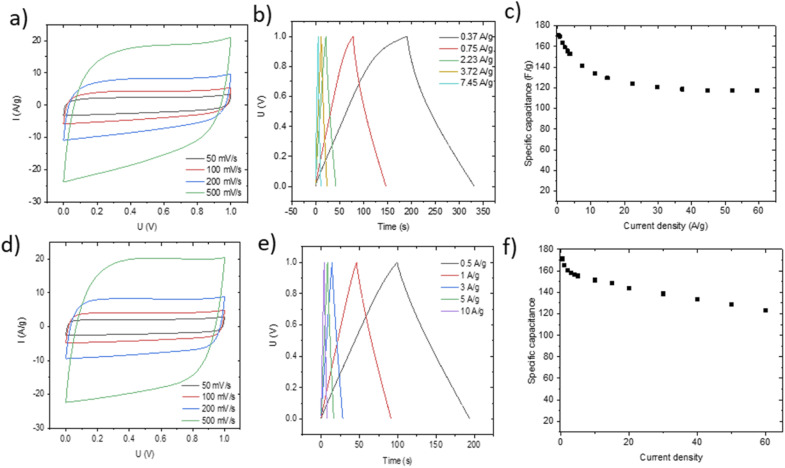
Electrochemical performance of electrodes prepared using PC-AC on stainless steel and tested in KOH electrolyte. Blade deposited electrodes (a–c) and pellet electrodes (total weight 13.6 mg)(d, e) Cyclic voltammetry (a and d), charge–discharge (b and e) and specific capacitance *vs.* current density plots (c and f).

The performance of blade deposited and pellet electrodes prepared using PC-AC was also verified in 6 M KOH electrolyte (Fig. 9) using stainless steel as a current collector.

Once again, the CV curves show a near square shape and with discharge curves close to linear. The Nyquist plots provide evidence for low cell resistance. The electrodes deposited from aqueous dispersions and pellet electrodes show a similar maximal gravimetric capacitance of 171 F g^−1^ at 0.5 A g^−1^ (Table 1) but somewhat different dependence on the current rate. Most likely, the behavior of electrodes at high current density is affected by difference in the components added to PC-AC (GO and fumed silica in dispersions; the PTFE binder in pellets).

**Table 1:** Summary for gravimetric capacitance recorded using PC-AC electrodes prepared by three methods in organic and aqueous electrolytes

Preparation method	Aqueous electrolyte (6 M KOH)		Organic electrolyte (1 M TEA-BF_4_)	
	Capacitance at 0.5 A g^−1^	Capacitance at 10 A g^−1^	Capacitance at 0.5 A g^−1^	Capacitance at 10 A g^−1^
Sprayed	—	—	105 F g^−1^	67 F g^−1^
Bladed	171 F g^−1^	141 F g^−1^	93 F g^−1^	73 F g^−1^
Pellet	171 F g^−1^	151 F g^−1^	90 F g^−1^	49 F g^−1^

**Fig. 10:**
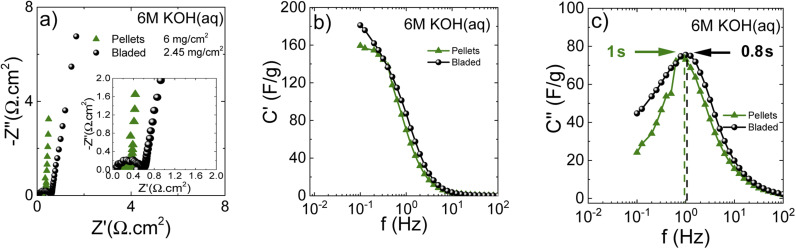
Impedance response of electrodes in aqueous KOH electrolyte. (a) Nyquist plots indicating a mass loading and a high-frequency region close-up; (b) real and (c) imaginary capacitance components according to the complex capacitance model showing the characteristic response time corresponding to the knee frequency.

The Nyquist plots (Fig. 10a) show insignificant differences between bladed and pelletized electrodes. This is because the residual oxygen in the electrode composition does not represent an important issue for the aqueous KOH electrolyte solution. Nevertheless, the close-up figure shows that the bladed electrode has some interface resistance. Further comparison using the real capacitance component (Fig. 10b) shows that both types of electrode lead to a similar frequency dependence of capacitance, showing that the cell performance in KOH electrolyte does not display a significant sensitivity to the electrode preparation method. Nevertheless, the higher mass loading of the pelletized electrodes results in a marginally steeper capacitance decay *vs.* frequency. This is also reflected in the characteristic response time (the imaginary capacitance plot in Fig. 10c), which is slightly higher for the pelletized electrodes.

**Fig. 11:**
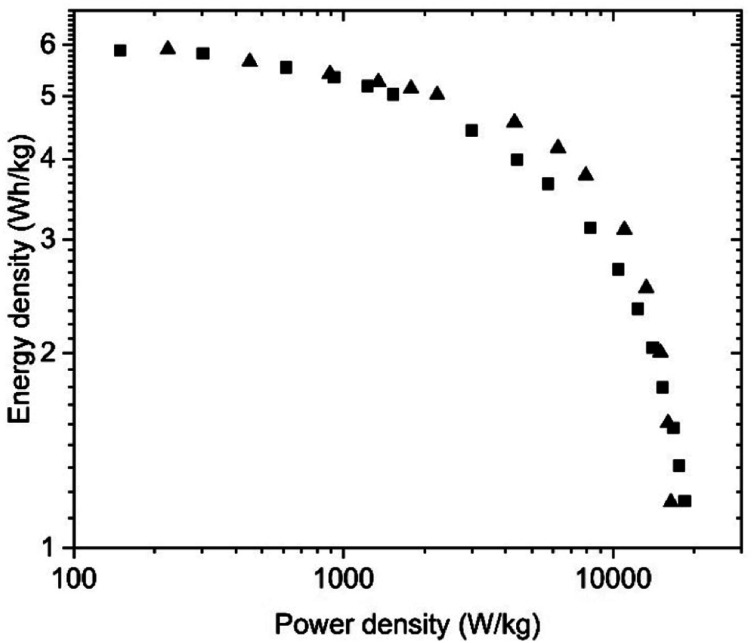
Ragone plots for the SC with PC-AC electrodes prepared using deposition from aqueous dispersions (blade) and (■) and pellet electrodes (▲) and tested in KOH electrolyte.

Once again, the energy storage parameters shown in the Ragone plots are rather similar for the SC with electrodes prepared using aqueous dispersions and pellets (Fig. 11). The somewhat higher energy density in the high-power region for the pelletized electrodes is associated with the higher overall resistance of the bladed electrodes as seen in the Nyquist plot above.

The PC-AC electrodes showed satisfactory stability upon cycling with retentions of 87.5% and 82% after 10 000 cycles for pellet electrodes and electrodes deposited from dispersions (respectively), Fig. S6, S7.†

Electrodes prepared using PC-AC were also tested using an ionic liquid, 1-ethyl-3-methylimidazolium tetrafluoroborate (EMI-BF_4_). Ionic liquids allow higher energy density due to the higher operating voltage. However, ionic liquids are low in electrolyte transport properties (viscosity and conductivity), which intrinsically limits the high-power performance. EMI-BF_4_ was chosen since it has one of the highest viscosities and conductivities known to date.

Fig. 11 shows a Nyquist plot in the IL electrolyte, revealing drastic differences in cell response depending on the electrode preparation method. These observations can be explained by the electrode texture and/or chemical composition, precluding the IL penetration into deeper pore positions. Thus, the lower contact resistance using the bladed electrodes can be due to the ionic liquid/carbon interface involving only a small part of the surface, mostly the outer pore surface, which ensures the lower overall contact resistance while simultaneously giving extended middle-and low-frequency regions due to the incomplete pore wetting with the IL.

Insufficient electrode wetting can be explained by the components used in the electrode preparation (such as rGO and fumed silica used in dispersions) and the electrode compaction method, which results in a poor compatibility with viscous IL electrolytes. At the same time, the pellets lead to a standard impedance response with a higher contact resistance due to more IL taking part in the interface, but also an almost ideal vertical-line capacitive response in the low-frequency range.

**Fig. 12:**
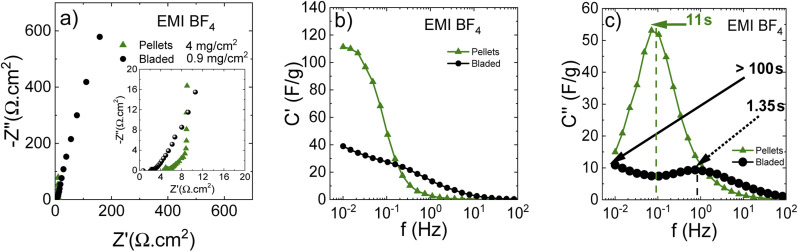
Impedance response of electrodes in solvent-free EMI BF_4_ electrolyte. (a) Nyquist plots indicating a mass loading and a high-frequency region close-up; (b) real and (c) imaginary capacitance components according to the complex capacitance model showing the characteristic response time corresponding to the knee frequency.

The real capacitance component (Fig. 12b) shows that the bladed electrodes present a lower capacitance decay for higher frequencies, but the capacitance value is also low, confirming the involvement of mostly the outer electrode surface. The pellet electrodes are associated with a higher capacitance decay, but also with a higher low-frequency capacitance resulting from the larger in-pore ion/carbon interface, as compared with a clearly underused potential contact surface area in the bladed electrodes. Remarkably, at frequencies above 0.2 Hz, the bladed electrodes deliver a higher capacitance than the pellets. This is likely due to the rGO being added to the bladed electrode composition, which makes a higher non-pore outer electrode surface that allows keeping some capacitive response at those frequencies where the capacitance at the low-external-surface pellets vanishes almost completely.

**Fig. 13:**
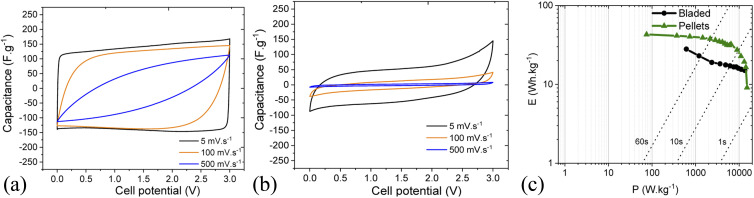
Rate performance in cyclic voltammetry for cells based on pellets (a) and bladed (b) electrodes using solvent-free EMI-BF_4_ electrolyte. (c) Ragone plot for bladed and pellet electrodes.

The imaginary capacitance component indeed shows two capacitive domains (Fig. 12c), the one with an extremely low time constant of 1.35 s (non-porous surface) while the other does not reach its maximum even at 10 mHz (100 s) due to the effective obstruction of the pore volume. Thus, it can be concluded that the IL electrolyte is incompatible with the porous carbon deposited by blading but can well work with low-surface materials intended for low-capacitance high-rate response. Fig. 13 demonstrates a rate performance under potentiodynamic conditions where the pellet electrodes respond to the higher scan rate with a typical voltammogram distortion related to an increased current-resistance contribution whereas the bladed electrodes rather display a drastic drop in capacitance as a function of scan rate. This observation agrees with the impedance data in that the electrolyte does not wet the inner pore surface and can be forced into it only under a relatively slow electrochemical stimulus of 50 mV s^−1^. At the higher rate, typically required from supercapacitors, only does the low rGO-related SSA provide some capacitance, which is also accordingly low (F g^−1^). The overall rate performance is reflected in a Ragone plot (Fig. 13c) showing that the pellets enable a maximum energy density of 45 W h kg^−1^ at a cell potential of 3 V, whereas the same is only 28 W h kg^−1^ for the bladed electrodes. However, the high-power region shows a more drastic drop in energy for the pellets, again due to their lower external surface in the pellet electrode composition since rGO is not part of the self-supported electrode composition, in line with the impedance results.

Summarising the results of electrochemical characterization (Table 1), the performance of PC-AC as electrodes in supercapacitors is in line with the high surface area and the essentially microporous nature of this material. For example, the gravimetric capacitance of coconut-based AC in KOH electrolyte was reviewed in a recent study by Keppetipola *et al.*,^7^ in the ∼100–260 F g^−1^ range for materials with A BET SSA of ∼1200–2900 m^2^ g^−1^. The data show no obvious trend for gravimetric capacitance *vs.* the BET SSA, similar to our earlier observation related to the performance of a-rGO in supercapacitors.^48^ The highest surface area is not always an advantage since it also leads to a lower density and volumetric capacitance and decreases the conductivity of electrodes. The microporous nature of materials might also limit the in-pore access of larger ions in organic electrolytes. At least part of the difference in performance can be explained by different procedures used to form electrodes and especially by the difference in the loading of materials.

It is also interesting to compare the performance of PC-AC and “activated graphene” prepared using the same KOH procedure. As expected from the very similar structure, porosity and surface area of these materials, supercapacitor electrodes also show similar gravimetric capacitance.^17,28,48^ For example, gravimetric capacitances of 185 F g^−1^ and 170 F g^−1^ were found for a-rGO and PC-AC in KOH electrolyte.^28^ A somewhat higher capacitance was also found for spray deposited a-rGO in TEA-BF_4_/ACN electrolyte, 120 F g^−1^ for a-rGO compared to 107 F g^−1^ for PC-AC.^28^ The spray deposition of PC-AC electrodes also appeared to be slightly favourable compared to the blade and pellet deposition methods (Table 1). Considering that PC-AC is produced from an essentially cost-free precursor (pine cones), it can be considered as an inexpensive alternative to “activated graphene.” In fact, one of the main results of our study is that “activated graphene” can be considered as a kind of activated carbon since using rGO or pine cone biochar as precursors provides nearly identical materials if the same KOH activation procedure is applied.

## Conclusions

In summary, pine cones were used as a naturally abundant precursor biomaterial for the preparation of activated carbon with an extremely high surface area of ∼3050 m^2^ g^−1^. The biochar produced by carbonization of pine cones was subjected to KOH activation previously optimized for activation of rGO and preparation of “activated graphene”. It is demonstrated that the same activation procedure applied to pine cone derived biochar results in an AC material with a surface area, pore volume and pore size distribution rather similar to “activated graphene” produced using rGO as a precursor. Therefore, it is reasonable to assume that “activated graphene” needs to be considered as a kind of activated carbon with only minor specific features related to the graphene (rGO) precursor. The PC-AC material was evaluated for preparation of supercapacitor electrodes using aqueous dispersions (spray and blade deposition on current collectors) and as free-standing pellets. It is demonstrated that aqueous dispersions based on PC-AC are compatible with spray gun deposition using large area current collectors. The energy storage parameters of electrodes deposited using the semi-industrial spray gun machine were comparable to electrodes prepared by laboratory scale methods.

The energy storage parameters of supercapacitors prepared using PC-AC electrodes appeared to be almost on the same level as “activated graphene” with a similar surface area. Therefore, using “green” and inexpensive pine cone derived AC can be considered as a feasible alternative to “activated graphene.” The biochar is obviously a lot easier to prepare by simple thermal carbonization whereas the cost of pine cones is negligible. In contrast, rGO is prepared starting from graphite using oxidation in concentrated acids to prepare GO and explosive thermal exfoliation of GO to prepare rGO with a high surface area. Pine cones are currently considered as a waste in the forest/wood industry. It is also an abundant precursor available in large amounts in industrial regions such as Europe (especially in Scandinavia), Canada and the USA.

## Conflicts of interest

There are no conflicts to declare.

## Supplementary Material

NA-004-D2NA00362G-s001
