# Supplementary material for: High-surface-area activated carbon from pine cones for semi-industrial spray deposition of supercapacitor electrodes
Source: Nanoscale Adv. 2022 Sep 21;4(21):4689–700. doi: 10.1039/d2na00362g (PMC9595184; doi:10.1039/d2na00362g)
Supplement: NA-004-D2NA00362G-s001 [file NA-004-D2NA00362G-s001.pdf]

Electronic Supplementary Material (ESI) for Nanoscale Advances.
This journal is © The Royal Society of Chemistry 2022

High-surface-area activated carbon from pine cones for semi-industrial spray deposition of supercapacitor electrodes.

Andreas Nordenström, Nicolas Boulanger, Artem Iakunkov, Gui Li, Roman Mysyk, Alexandr Talyzin

[a] *Department of Physics, Umeå University, Umeå, Sweden c IKERBASQUE, Basque Foundation for Science, 48013 Bilbao, Spain*

[b] *Centre for Cooperative Research on Alternative Energies (CIC energiGUNE), Basque Research and Technology Alliance (BRTA), Alava Technology Park, Albert Einstein 48, 01510 Vitoria-Gasteiz, Spain*

1. Electrochemical characterization of supercapacitors. Details of calculations.

Specific capacitance, energy density and power density were estimated based on the galvanostatic discharge data using the standard procedure reported in literature (see details of calculations in SI file) [45].

The capacitance was determined from galvanostatic constant current charge/discharge curves measured at different current densities. For each discharge cycle, integration was performed starting not from the highest voltage (U_{Max}) but from a slightly lower voltage (U_{Start}). The reason for this is that there is a voltage drop from U_{Max} to U_{Start} as a result of Ohmic resistance in the cell, which should not be included in capacitance calculations.

The total capacitance of the electrodes in the SC setup (C_{total}) is given by

$$C_{total} = 2 * I * \frac{\int_{U_{Min}}^{U_{Start}} U dt}{\left(U_{Start}^2 - U_{Min}^2\right)}, \quad (1)$$

where $\int U dt$ is the integrated area under the discharge curve, I is the applied current and U_{Min} is the voltage corresponding to the end point of the integrated area. Note that we used the convention to measure the capacitance when the electrodes are fully discharged, meaning that $U_{Min} = 0$ V, which may differ from other common operating conditions (usually $U_{min} = 0.5U_{max}$), which are compatible with AC/DC converters in electric circuitries needed for common use under constant-voltage conditions.

The energy density E of the electrodes can be determined for each charge/discharge as

$$E = \frac{1}{M} I \int_{U_{Min}}^{U_{Start}} U(t) dt = \frac{1}{M} \frac{1}{2} C_{total}\left(U_{Start}^2 - U_{Min}^2\right), \quad (2)$$

The power density P can be calculated from the energy density by

$$P = \frac{E}{\Delta t}, \quad (3)$$

where Δt is the time of discharge in seconds.

Impedance spectroscopy was conducted using frequencies down to 0.1 Hz for aqueous and organic electrolytes and 0.01 Hz for the ionic liquid electrolyte.

Complex capacitance *C(ω)* was calculated from the following equations [46]:

$$C(\omega) = C'(\omega) - jC''(\omega) \quad (4)$$

$$C'(\omega) = \frac{-Z''}{|Z|^2\omega} \qquad\qquad C''(\omega) = \frac{Z'}{|Z|^2\omega} \qquad (5)$$

where $C'(\omega)$ and $C''(\omega)$ are the real and imaginary capacitance components, Z, Z' and Z'' the total, real and imaginary resistance, ω the radial frequency *(ω=2πf)*.

2. Additional characterization of PC-AC

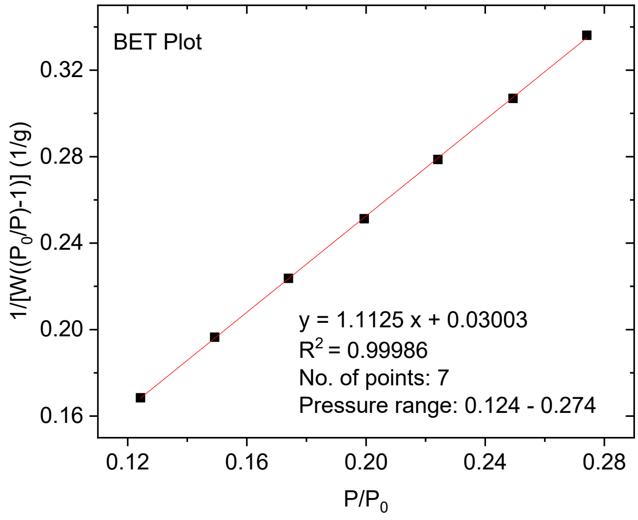

Figure 1S. Example BET plot used to estimate BET SSA of PC-AC sample. The relative pressure range was determined using procedure based on Rouquerol parameters available as a part of standard software package provided by Quantachrome instrument manufacturer. The BET surface values were also verified using BETSI software (**Table1**) which provides very similar value using 11 points with somewhat broader range of relative pressures and little smaller R^2 value of 0.9956. It is our opinion that cumulative surface area provided by DFT provides values of surface area closer to real is best to be used to compare microporous samples. The surface area determined by DFT is more reproducible and not affected by somewhat subjective procedures typical for BET method.

Table 1S. Values of specific surface area determined using automatic procedure by Quantachrome ASiQWin, DFT slit pore model and BETSI software.

	BET SSA	Cumulative SSA	BETSI SSA*
**Batch 1**	2847	2116	2847 (7)
**Batch 2**	3047	2249	3049 (8)
**Batch 3**	3048	2280	3049 (7)

* SSA calculate using BETSI software when the program selects optimal number of points > 3. Number in parenthesis indicate how many points were used in the BET plot.

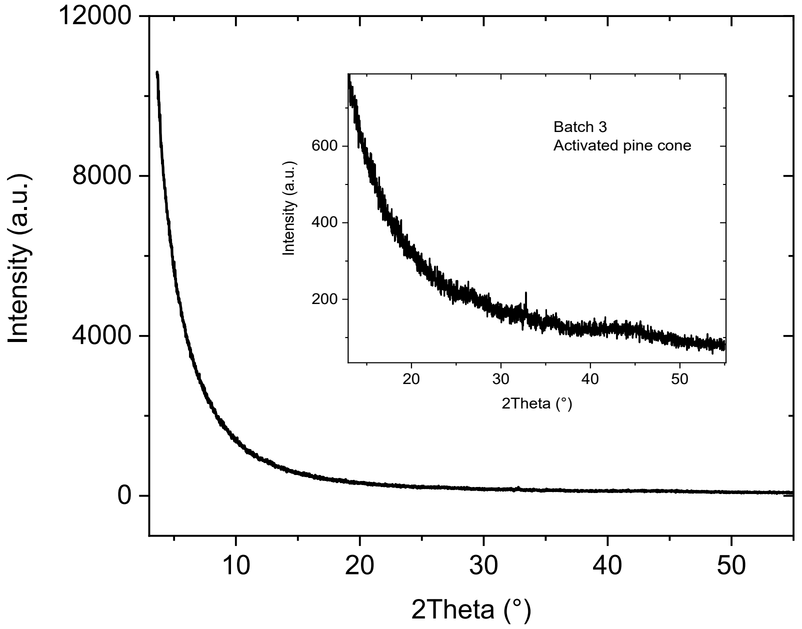

Figure S2. XRD pattern of PC-AC recorded from sample Batch 3 in the **Table 1S**. Note the signal from diffuse scattering in the low angle region (below ~10 degrees). Inset shows the part of pattern where XRD reflections from graphitic carbon are expected to appear. The structure of PC-AC is completely disordered as expected for high surface area porous carbon materials.

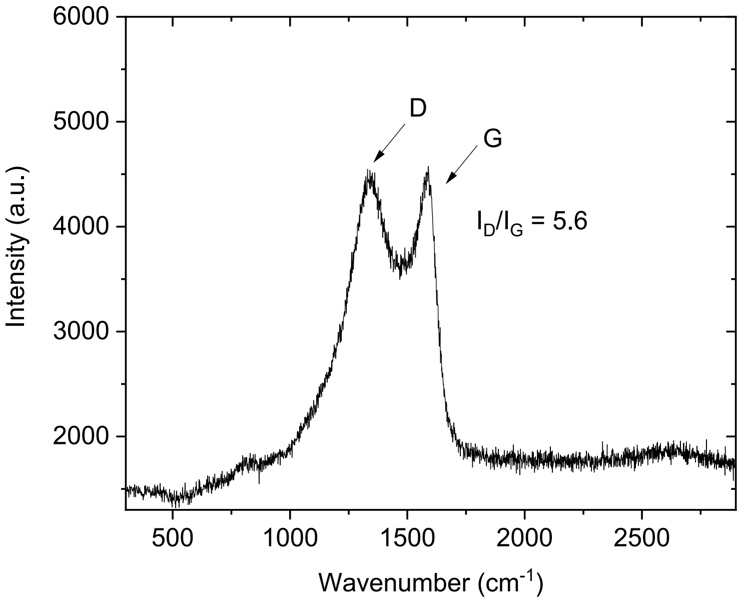

Figure S3. Raman spectrum recorder from PC-AC sample. Notably the G-mode is broader than D mode providing I_D/I_G ratio of 5.6.

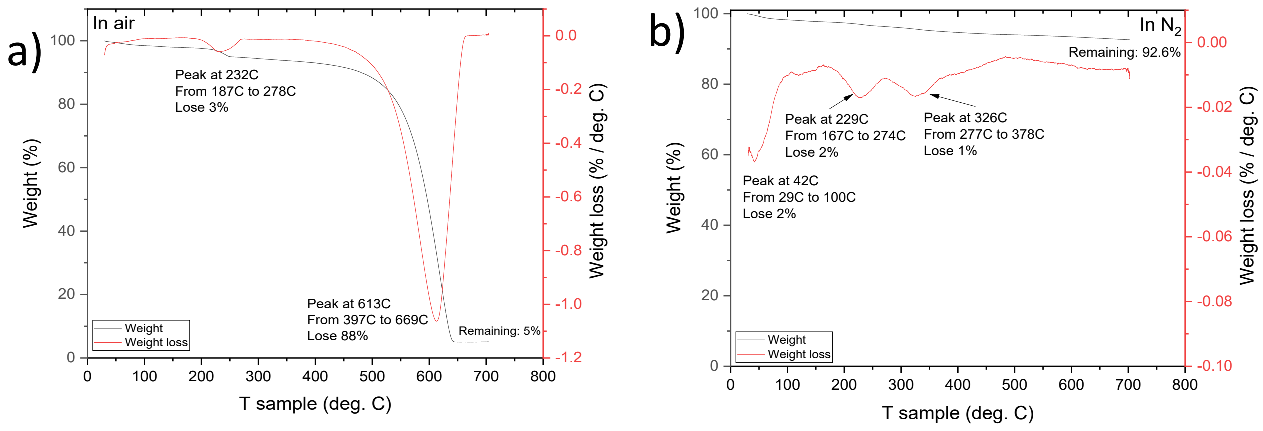

Figure S4. TGA data for PC-AC sample with C/O=10.1 according to XPS. a) recorded under air and b) recorded under nitrogen. The sample has relatively high 5% residue which originates from Al2O3 contamination. As a result this sample has somewhat smaller surface area and

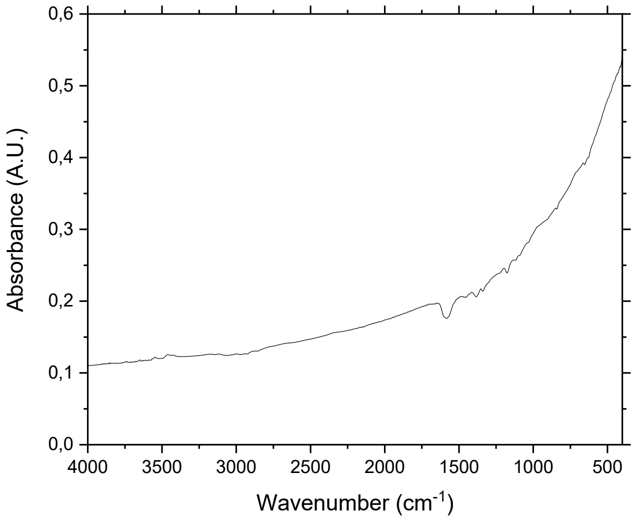

Figure S5. FTIR spectrum of PC-AC showing strong non linear background and few weak features typical for AC materials.

Table S2. Conductivity tested using strips composed by AC material mixed with binder and electrodes spray deposited using standard formulation of dispersion which include AC:GO:SiO2:CNT in 10:1:1:1 proportion.

Conductivity (S/m)						
Materials	Ratio with binder	Strip 1	Strip 2	Strip 3	Strip 4	Average
AC strip	8.16:1	0.12	0.14	/	/	**0.13**
PC-AC Electrode	10:1:1:1	61.7	41.0	68.9	45.8	**54.4**

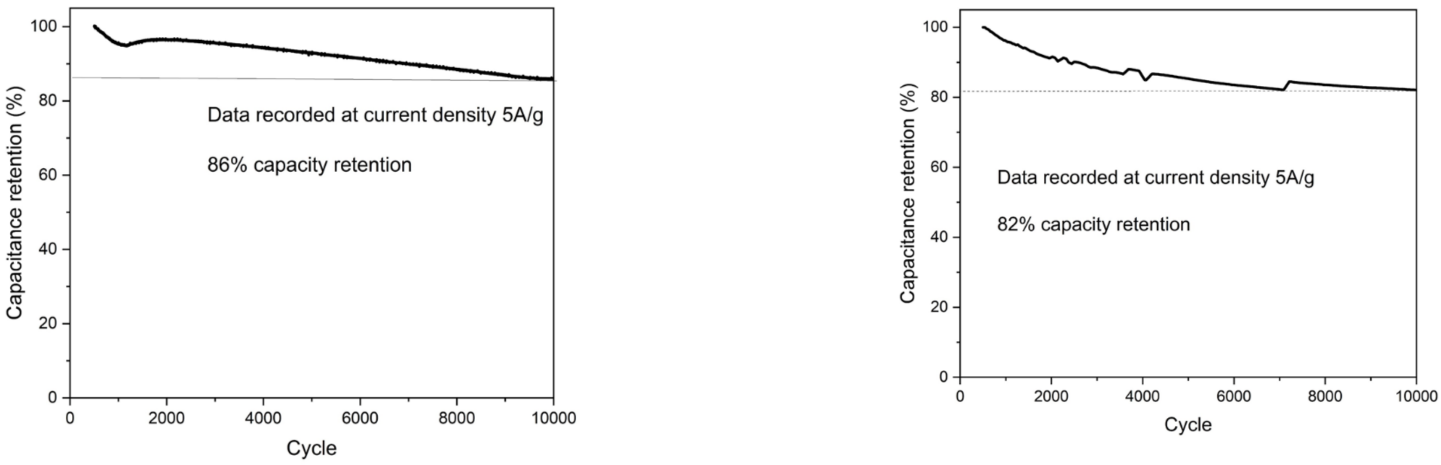

a) b)

Figure S6. Cycling performance of PC-AC electrodes in KOH electrolyte recorded at 5A/g current density: a) pellet electrodes, b) blade deposited electrodes.

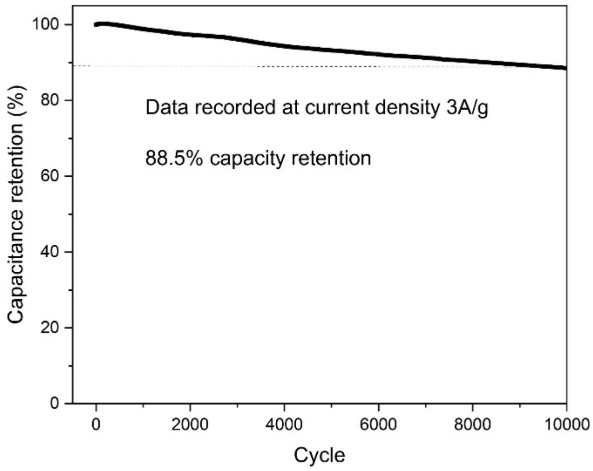

Figure S7 Cycling performance of PC-AC electrodes in TEA-BF4 CAN electrolyte recorded at 3A/g current density: pellet electrodes
